# Fasting inhibits colorectal cancer growth by reducing M2 polarization of tumor-associated macrophages

**DOI:** 10.18632/oncotarget.20301

**Published:** 2017-08-16

**Authors:** Pengfei Sun, Huihui Wang, Zhiyong He, Xiangyuan Chen, Qichao Wu, Wankun Chen, Zhirong Sun, Meilin Weng, Minmin Zhu, Duan Ma, Changhong Miao

**Affiliations:** ^1^ Department of Anesthesiology, Fudan University Shanghai Cancer Center, Department of Oncology, Shanghai Medical College, Fudan University, Shanghai, China; ^2^ Key Laboratory of Metabolism and Molecular Medicine, Ministry of Education, Department of Biochemistry and Molecular Biology, Collaborative Innovation Center of Genetics and Development, Institutes of Biomedical Sciences, School of Basic Medical Sciences, Fudan University, Shanghai, China

**Keywords:** fasting, tumor-associated macrophages, colorectal cancer, adenosine, autophagy

## Abstract

Dietary restriction has been recognized as a healthy and natural therapy for cancer. It is reported that different forms of dietary restriction can promote anti-tumor immunity. However, it is not clear how fasting affects tumor-associated macrophages (TAMs). This study aims to investigate the relationship between fasting and antitumor immunity in terms of tumor-associated macrophages. *In vivo,* the results showed that alternate day fasting for 2 weeks inhibitted the tumor growth of mice without causing a reduction of body weight. Meanwhile, M2 polarization of tumor-associated macrophages in tumor tissues of alternate day fasting group was also decreased. *In vitro*, fasting induced the autophagy of CT26 cells, decreased the generation of extracellular adenosine by supressing the expression of CD73 in CT26 cells. Decreasing adenosine inhibitted M2 polarization of RAW264.7 cells through inactivating JAK1/STAT3 signal pathway in fasting condition. Eventually, the proliferation of CT26 cancer cells declined on account of fasting-facilitated antitumor immunity. These results suggested that fasting suppressed M2 polarization of tumor-associated macrophages to inhibit tumor growth through decreasing the level of adenosine in the tumor microenvironment both *in vivo* and *in vitro*. This process was associated with increasing autophagy of tumor cells.

## INTRODUCTION

The morbidity and mortality of colorectal cancer is increasing year by year, which threatens human health severely. It is generally believed that colorectal cancer is associated with high fat, high carbohydrate and high protein diet [1, 2]. Obesity is also considered to be a risk factor for colorectal cancer [3, 4]. So it is necessary to investigate the relationship between dietary intervention and colorectal cancer.

Interactions between cancer metabolism and immunity are arousing wide concern recently. Preliminary data indicates that short-term fasting or ketogenic diet, both to induce physiological hungry state, can reverse immunosuppressive character of the tumor microenvironment through decreasing lactic acid or increasing ATP in the tumor microenvironment [5, 6]. Most of these studies focus on immune cells such as regulatory T cells (Tregs), myeloid-derived suppressor cells (MDSCs), or NK cells [5, 6]. Meanwhile however, few studies are reported about fasting and tumor-associated macrophages, which are among the most important immune components of tumor microenvironment.

Tumor-associated macrophages (TAMs) possess type M2 macrophage biology characteristics and play a critical role in immunity in the tumor microenvironment. It is generally accepted that TAMs can shield tumor immune surveillance and stimulate new blood vessel formation, induce proliferation of cancer cells, facilitate local invasion and distant metastasis of tumor cells [7, 8].

Here, using both *in vitro* and *in vivo* colon carcinoma models, we found that fasting could suppress M2 polarization of TAMs through inactivating JAK1/STAT3 in the tumor microenvironment and therefore inhibit tumor cell growth. This process involved the lower generation of extracellular adenosine in the tumor microenvironment through increased cancer cell autophagy and decrease of CD73, an ectoenzyme that degrades extracellular ATP into immunosuppressive adenosine. Our study highlighted the importance of fasting in inhibiting colorectal cancer growth through lowering generation of adenosine and suppressing M2-TAMs proliferation in the tumor microenvironment.

## RESULTS

### Fasting inhibits tumor growth both *in vivo* and *in vitro*

**Figure 1:**
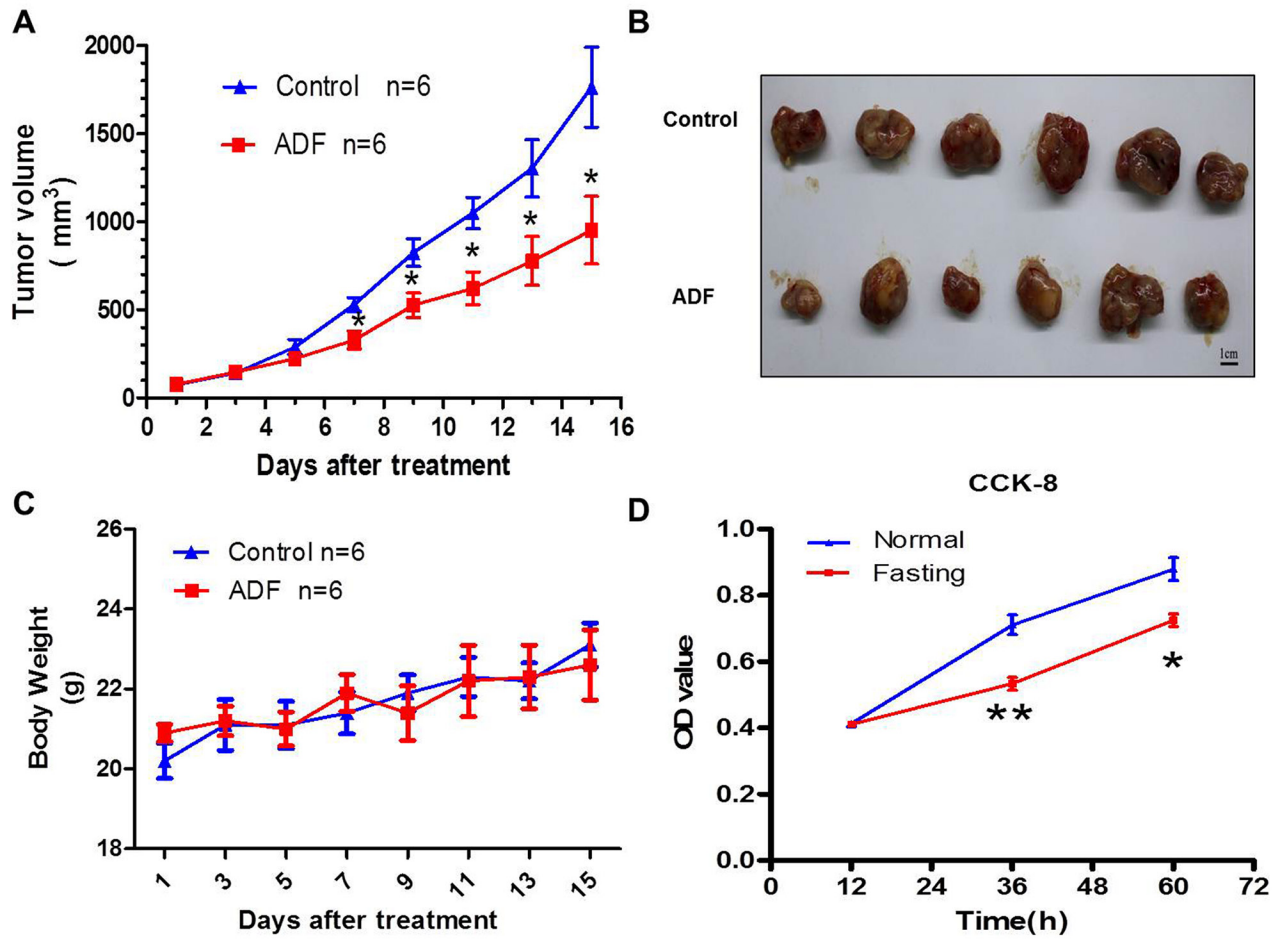
Fasting inhibits tumor growth both *in vivo* and *in vitro* **(A)** Tumor growth curve of mice from Control and ADF groups for 2 weeks. *P<0.05. **(B)** Subcutaneously implanted tumors dissected from mice of Control and ADF groups. **(C)** Body weights of mice from Control and ADF groups. Data shows no significant difference between the two groups. **(D)** The proliferation of CT26 cells in normal or fasting coculture condition *in vitro* was tested by CCK8. *P<0.05, **P<0.01.

Mice colorectal cancer model was constructed in this study. Compared with mice in control group, the tumor growth of ADF group was suppressed (Figure 1A, 1B), without a reduction in body weight (Figure 1C). For *in vitro* experiments, CT26 cancer cells and RAW264.7 macrophage cells were cocultured in fasting or normal RPMI-1640 medium. We found that the proliferation of CT26 cells was suppressed in fasting medium (Figure 1D).

### Fasting suppresses M2 polarization of TAMs both *in vivo* and *in vitro*

**Figure 2:**
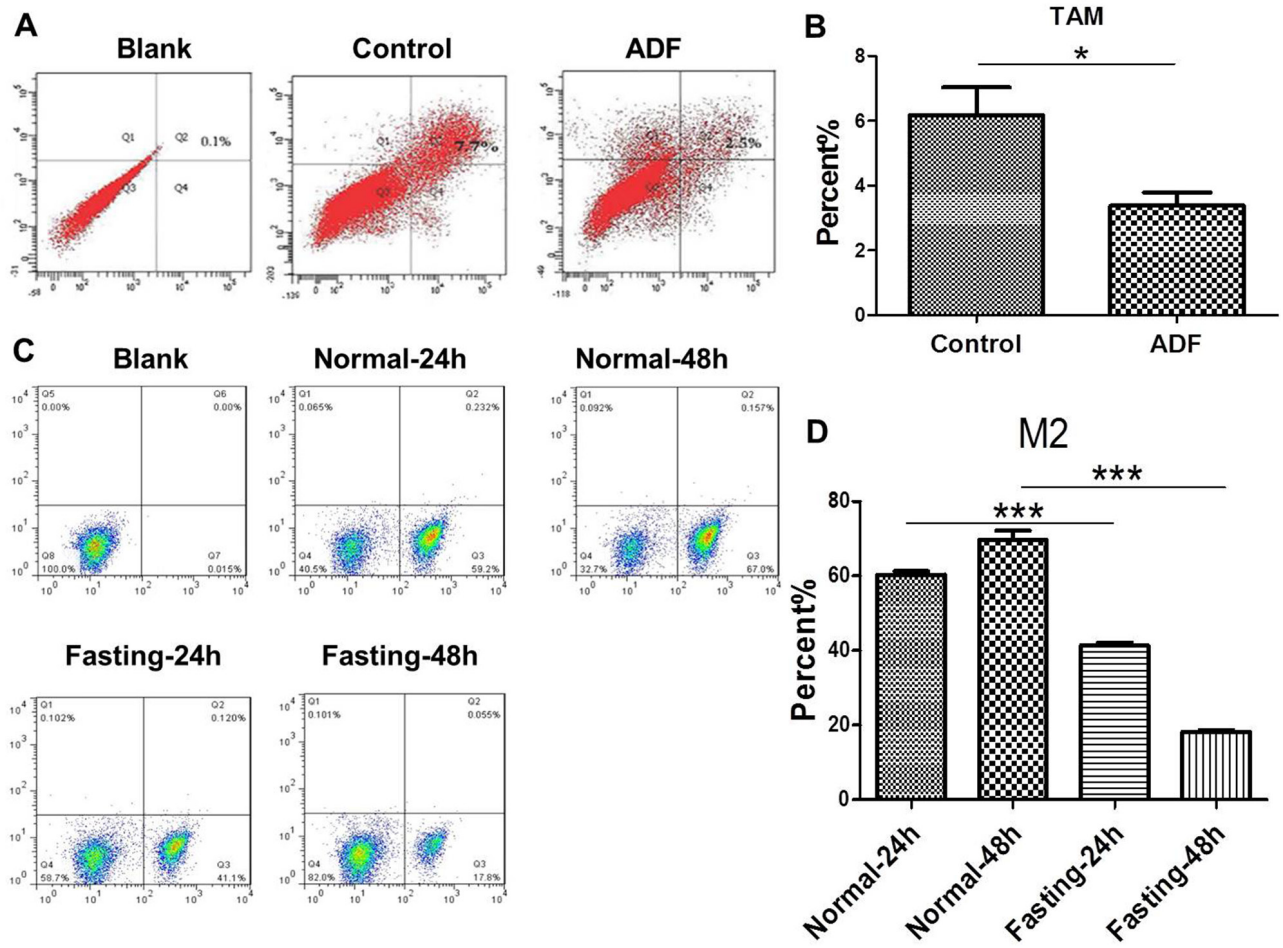
Fasting suppresses M2 polarization of TAMs both *in vivo* and *in vitro* **(A-B)** The levels of M2 macrophages were detected by FCM (flow cytometry) in tumor tissues of mice from Control and ADF groups (ADF: alternate day fasting). *P<0.05. **(C-D)** The levels of M2 macrophages were detected by FCM (flow cytometry) in normal or fasting medium for 24 h or 48 h. ***P<0.001.

In order to find out whether fastinginhibits tumor growth through modulating TAMs, we tested the M2 polarization of TAMs. For *in vivo* experiments, we found that M2 polarization of TAMs was reduced in tumor tissues of ADF group (Figure 2A, 2B). For *in vitro* experiments, CT26 cancer cells and RAW264.7 macrophage cells were cocultured in fasting or normal RPMI-1640 medium for 24 h or 48 h. The results of flow cytometry showed that M2 polarization of RAW264.7 cells was reduced in fasting condition (Figure 2C, 2D).

### Fasting induces cancer cell autophagy, downregulates CD73 expression of cancer cells and suppresses adenosine generation *in vitro*

**Figure 3:**
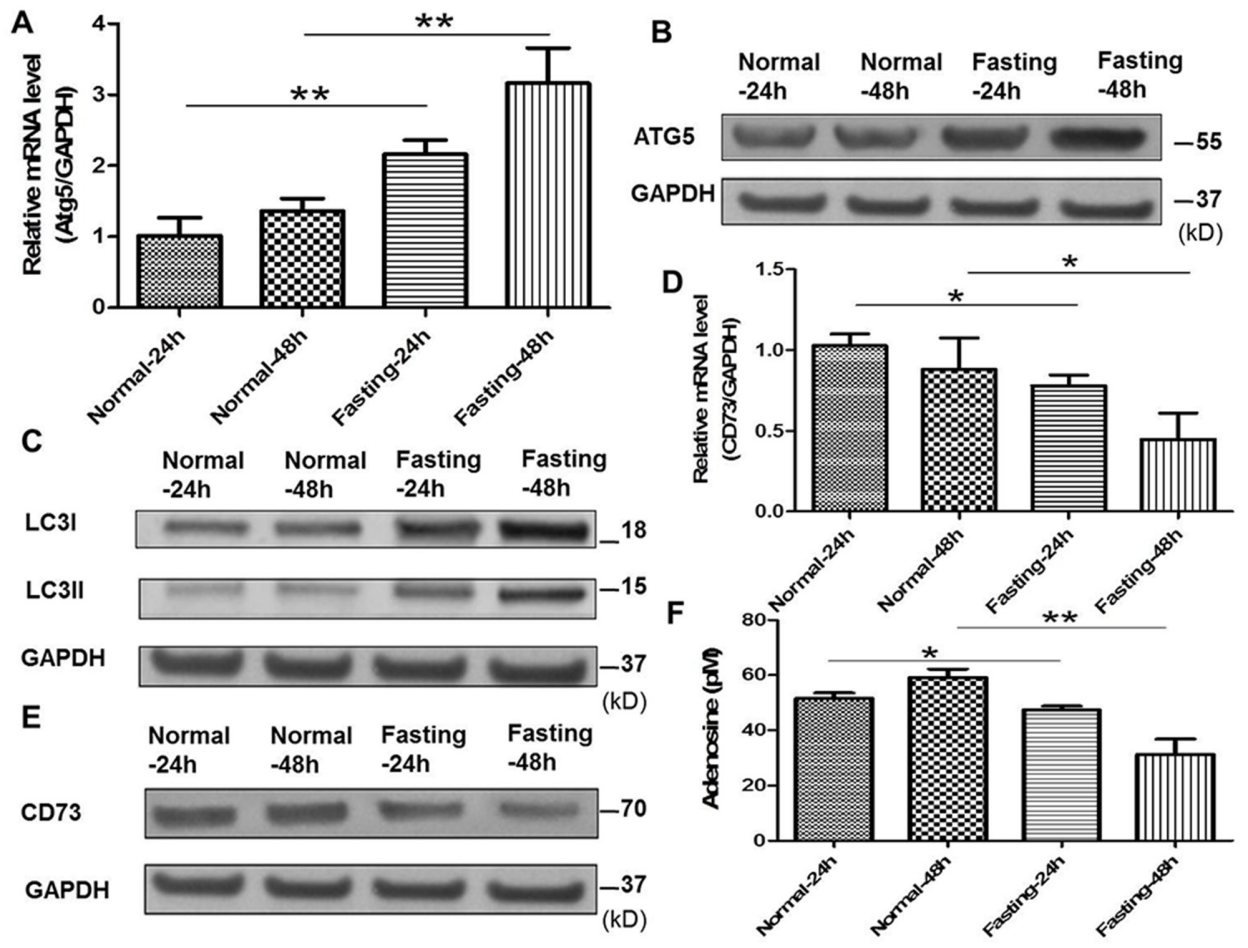
Fasting induces cancer cell autophagy, downregulates CD73 expression of cancer cells and suppresses adenosine generation *in vitro* **(A)** The mRNA expression of Atg5 was examined by qPCR in normal or fasting medium for 24 h or 48 h. **P<0.01. **(B)** The expression of Atg5 was tested by Western blotting. **(C)** The expression of LC3I/II was tested by Western blotting. **(D)** The mRNA expression of CD73 was tested by qPCR. *P<0.05. **(E)** The expression of CD73 was determined by Western blotting. **(F)** The levels of adenosine were tested by ELISA. *P<0.05, **P<0.01.

Previous studies have reported that autophagy of tumor cells can downregulate the expression of ectoenzymes such as CD39 and CD73 related to adenosine generation [9, 10]. Atg5 and LC3II/I were the indicators for the autophagy of cancer cells. In our study, the expression of Atg5, LC3II/I and CD73 in CT26 cells with fasting or normal medium was detected by qPCR or Western blotting. The results showed that fasting increased the expression of Atg5 and LC3II/I (Figure 3A, 3B, 3C) and decreased the expression of CD73 in CT26 cells (Figure 3D, 3E). Moreover, the generation of adenosine was reduced (Figure 3F).

### Knockdown of Atg5 in cancer cells enhances adenosine’s effect on macrophages and tumor growth *in vitro*

**Figure 4:**
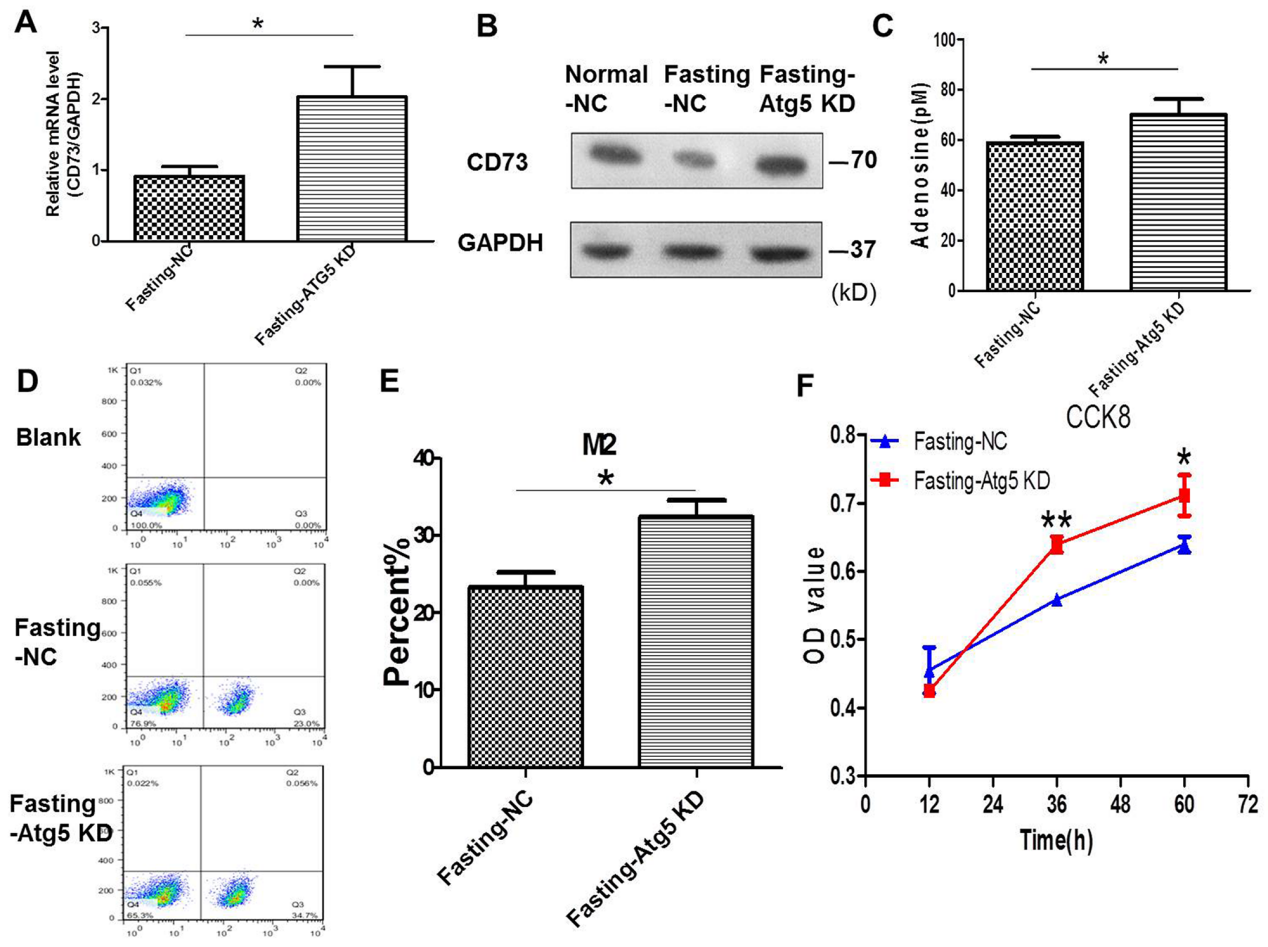
Knockdown of Atg5 in cancer cells enhances adenosine’s effect on macrophages and tumor growth in coculture condition **(A-F)** CT26 cells were stably transfected with shAtg5 (Atg5 KD) or shNC (NC). (A-B) The CT26 expression of CD73 was detected by qPCR in fasting medium. *P<0.05. (C) The levels of extracellular adenosine were tested by ELISA. *P<0.05. (D-E) The levels of M2 macropages were detected by FCM (flow cytometry). *P<0.05. (F) The proliferation of CT26 cells was tested by CCK8. *P<0.05, **P<0.01.

To verify the role of autophagy in this study, autophagy-related Atg5 of CT26 cells was knocked down by Atg5 shRNA (Supplementary Figure 1) in fasting medium. We found that the expression of CD73 (Figure 4A, 4B) and the levels of extracellular adenosine (Figure 4C) were enhanced after knockdown of Atg5 in CT26 cells in the coculture system for 24 h. The M2 polarization of macrophages (Figure 4D, 4E) and the proliferation of CT26 cells were increased simultaneously (Figure 4F).

### Knockdown of CD73 in cancer cells suppresses adenosine’s effect on macrophages and tumor growth *in vitro*

**Figure 5:**
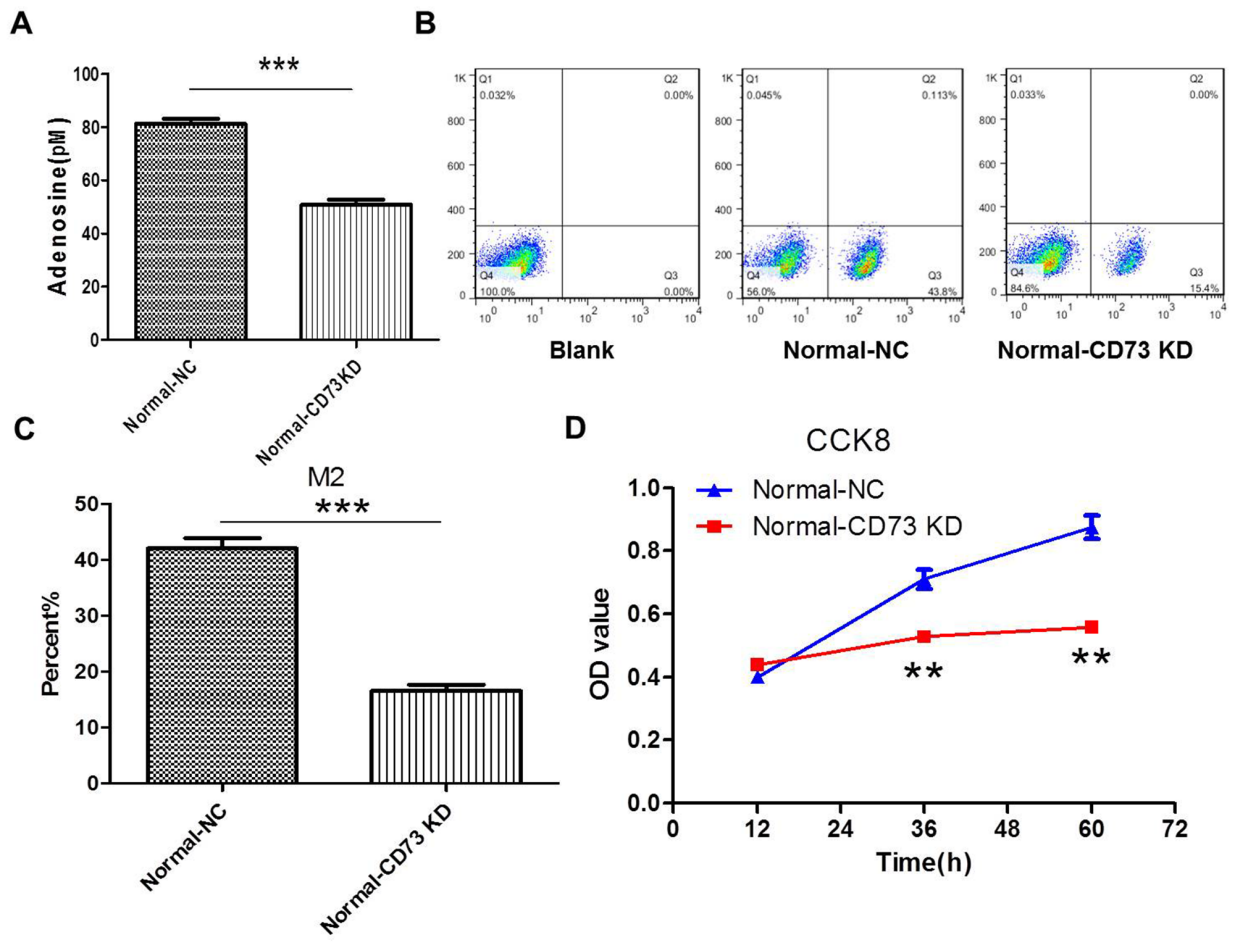
Knockdown of CD73 in cancer cells suppresses adenosine’s effect on macrophages and tumor growth in coculture condition **(A-D)** CT26 cells were stably transfected with shCD73 (CD73 KD) or shNC(NC). (A) The levels of extracellular adenosine were tested by ELISA in normal medium. ***P<0.001. (B-C) The levels of M2 macropages were detected by FCM (flow cytometry). ***P<0.001. (D) The proliferation of CT26 cells was tested by CCK8. **P<0.01.

We postulated that CD73 contributed to this progression by regulating adenosine generation. So in this part, CD73 of CT26 cells was knocked down by CD73 shRNA (Supplementary Figure 2). The results showed that the levels of extracellular adenosine were decreased after knockdown of CD73 in CT26 cells in the coculture system for 24 h. Accordingly, the polarization of M2 macrophages (Figure 5A, 5B, 5C) and the proliferation of CT26 cells were inhibitted (Figure 5D).

### PSB1115 treatment downregulates adenosine’s effect on macrophages and tumor growth *in vitro*

**Figure 6:**
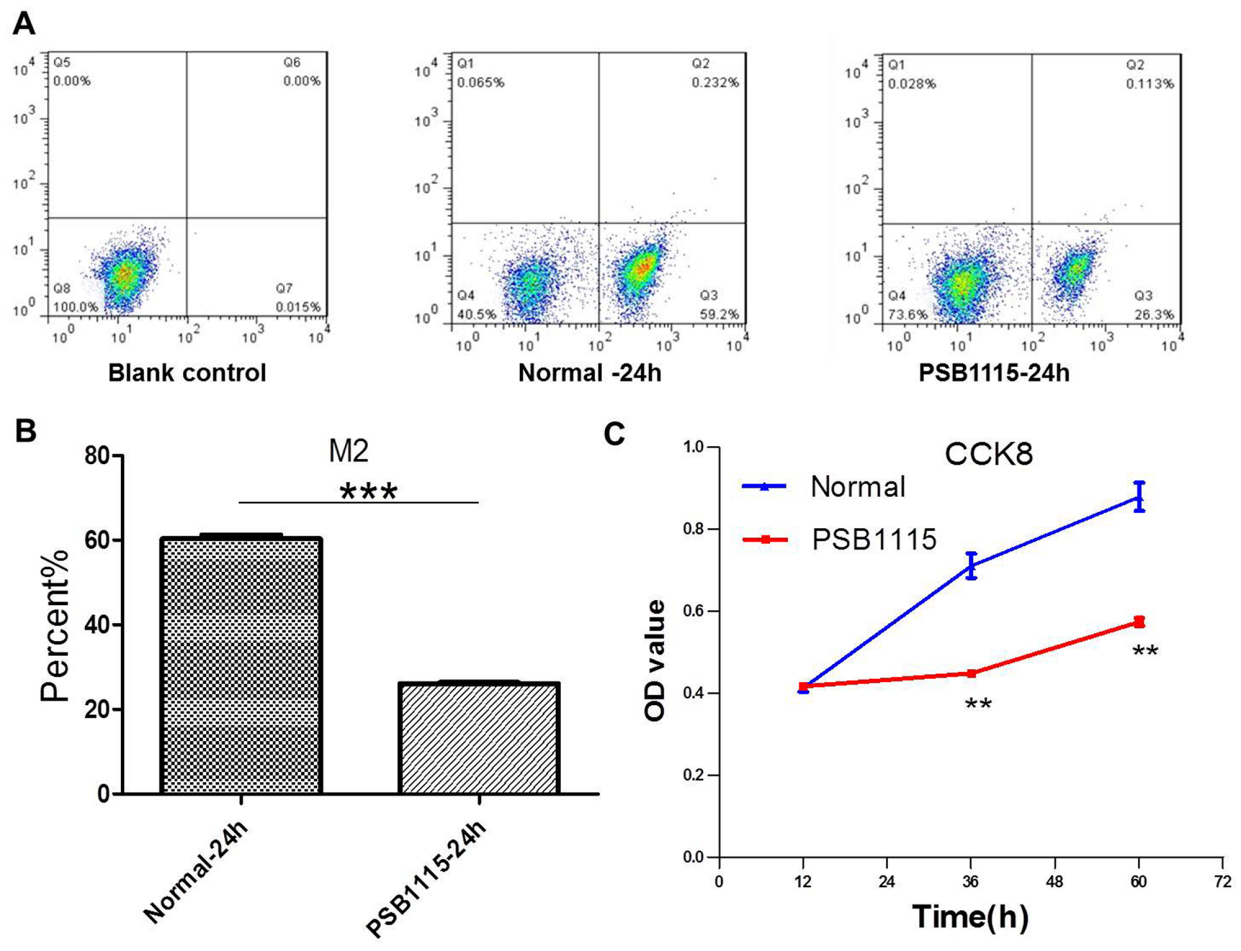
PSB1115 treatment downregulates adenosine’s effect on macrophages and tumor growth in coculture condition **(A-B)** The levels of M2 macropages were detected by FCM (flow cytometry) in normal medium with or without PSB1115 treatment for 24 h. ***P<0.001. **(C)** The proliferation of CT26 cells was tested by CCK8. **P<0.01.

It is reported that adenosine, as an immuno-suppressive endogenous purine nucleoside with high expression in the tumor microenvironment, can induce M2 polarization of TAMs [11, 12]. So in this part of study, the antagonist PSB1115 was used to block the binding of adenosine to its receptors of macrophages at a concentration of 1 μM. The M2 polarization of macrophages (Figure 6A, 6B) and the proliferation of CT26 cells (Figure 6C) were suppressed after PSB1115 treatment for 24 h in coculture system.

### Fasting suppresses M2 polarization of macrophages through inactivating JAK1/STAT3 signal pathway

**Figure 7:**
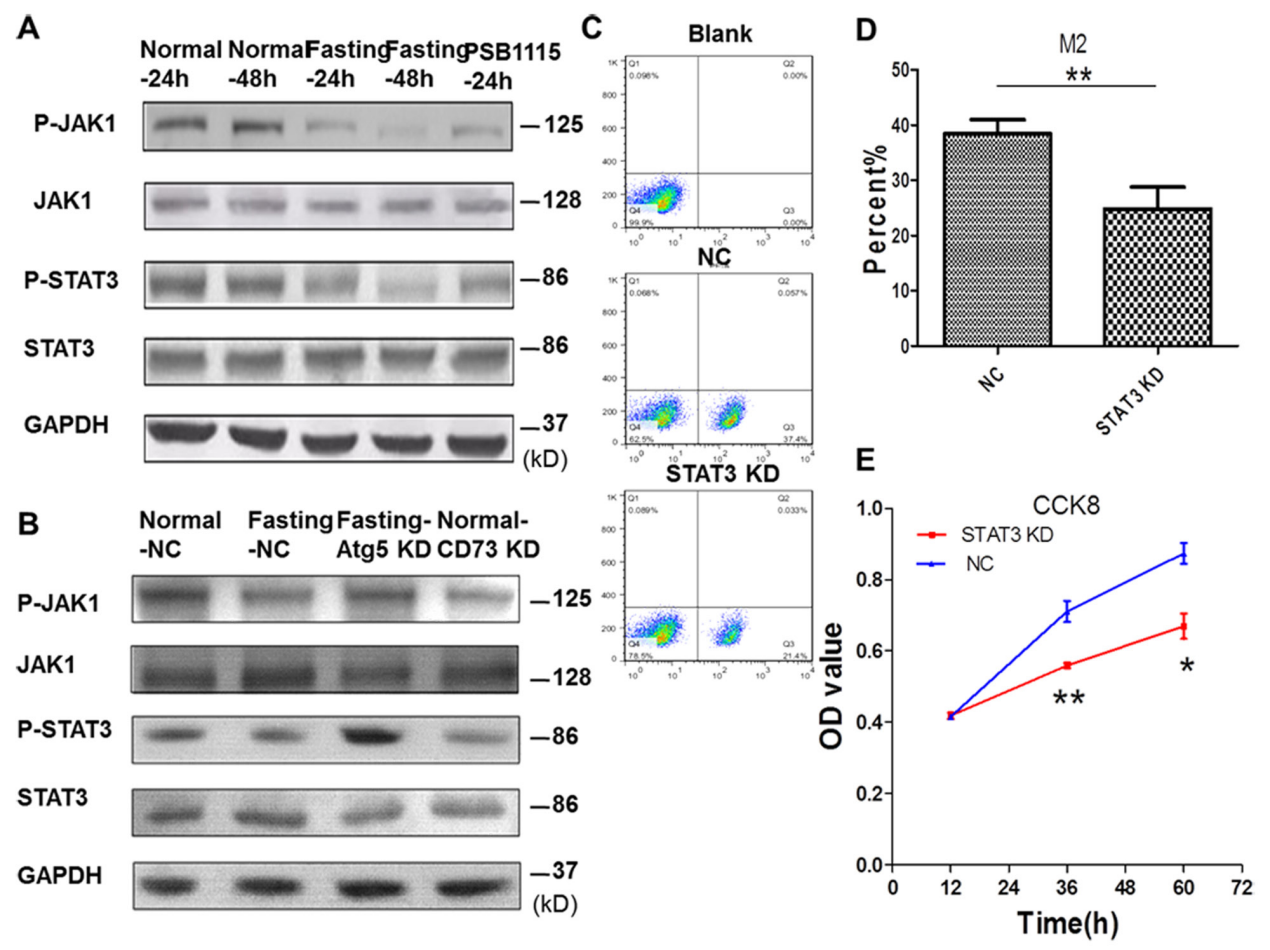
Fasting suppresses M2 polarization of macrophages through inactivating JAK1/STAT3 signal pathway in coculture condition **(C-E)** Raw264.7 cells were stably transfected with shSTAT3 (STAT3 KD) or shNC (NC). **(A)** The expression of JAK1/STAT3 signal pathway of RAW264.7 cells was determined by Western blotting. **(B)** The expression of JAK1/STAT3 signal pathway of RAW264.7 cells was determined by Western blotting when Atg5 or CD73 of CT26 cells were knocked down. (C-D) The levels of M2 macropages were detected by FCM (flow cytometry). **P<0.01. (E) The proliferation of CT26 cells was tested by CCK8. *P<0.05, **P<0.01.

To find out the mechanism involved in the suppression of M2 polarization of TAMs by fasting, we investigated the expression of JAK1/STAT3 signal pathway in TAMs. For *in vitro* experiments, CT26 cells and RAW264.7 cells were cocultured for 24 h or 48 h. The results showed that the phosphorylation of JAK1 and STAT3 in RAW264.7 cells in fasting condition was suppressed (Figure 7A). Knockdown of CD73 in CT26 cells (Figure 7B) or PSB1115 treatment in normal coculture condition also inhibitted the phosphorylation of JAK1 and STAT3 in RAW264.7 cells. The inactivation of JAK1/STAT3 signal pathway of RAW264.7 cells was reversed after Atg5 of CT26 cells was knocked down in fasting coculture condition (Figure 7B). Knockdown of STAT3 in RAW264.7 cells in normal cocluture system (Supplementary Figure 3) suppressed M2 polarization of RAW264.7 cells and proliferation of CT26 cells (Figure 7C, 7D, 7E).

## DISCUSSION

Under nutrition deprivation caused by fasting, tumor cells have higher levels of autophagy [13, 14]. It is believed that autophagy plays dual roles in the development of cancer by activating the immune response in some cases, and impairing the anti-tumor immunity in others. It is reported that in early oncogenesis, autophagy is often inhibitted. However, at advanced stage, autophagy is required for the progression of cancers [15, 16], suggesting that inducing autophagy in early stages of tumor progression may be an effective way to promote anti-tumor immunity. Of note, nutrition starvation is one of the most efficient ways to elicit autophagy in most cells [13, 17]. Moreover, both fasting and autophagy are reported to promote rejuvenation and delay senescence of hematopoietic stem cells so as to maintain the function of immune system [9, 18]. In our study, mice were subjected to alternate-day fasting when subcutaneous tumors formed. Without a reduction in body weight, the alternate-day fasting mice were as active and healthy as the AL fed mice and tumor sizes were reduced compared with control group. Our results showed that in this stage of CT26 tumor progression, fasting could promote anti-tumor immunity by inducing tumor autophagy. Our work provided a new evidence for the complex interactions among autophagy, tumor immunity and tumor progression.

It is reported that autophagy decreases expression of ectoenzymes such as CD39 and CD73 in lung cancer and reduces levels of adenosine [10, 14]. Here we found that autophagy in CT26 tumor cells decreased CD73 expression. As some reports suggest that HIF-1α could increase the expression of genes encoding CD39 and CD73 [13, 19], whether fasting-induced autophagy reduces the level of CD73 through decreasing HIF-1α needs further study.

Adenosine is well known to be an immuno-suppressive endogenous purine nucleoside with high expression in the tumor microenvironment [11, 12]. Macrophages are the major component within the tumor microenvironment (usually termed TAMs) participating both in the progression of tumor growth and suppression of antitumor immunity. TAMs exhibit a predominantly M2-like phenotype [20, 21]. Our work further verified the role of adenosine in suppressing anti-tumor immunity by linking it to fasting and TAMs.

Adenosine can bind to both A2A and A2B receptors, mainly A2B receptors [22, 23], on the membrane of TAMs to increase the M2 polarization. Our work used A2B receptor antagonist PSB1115 and found that A2B receptors were involved in adenosine’s effect on TAMs polarization. It is reported that A2B receptors worked at a high concentration of adenosine [24]. However, our study found that A2B receptor blockade could reduce M2 polarization of TAMs at a relatively low concentration of adenosine. Our further study will investigate the role of A2A receptors in this process.

The therapeutic potential of A2B receptor blockade has been reported [25–28]. To our knowledge, this is the first study demonstrating that selective blockade of A2B receptor by PSB1115 can be effective in delaying colon cancer growth by inhibiting TAMs accumulation and restoring antitumor immunity.

We also explored the relevant signal pathways associated with adenosine regulating of M2 polarization of TAMs. There are different signal pathways involved in polarization of macrophages such as MAPK/JNK, PI3K/AKT and JAK/STAT [29–31]. Here we found that JAK1/STAT3 signal pathway played an important role in M2 polarization of TAMs. We used PSB1115 to block adenosine from binding to its receptors on the surface of macrophages, and found that the phosphorylation of JAK1 and STAT3, M2 polarization in RAW264.7 cells were inhibitted, and the proliferation of CT26 cells was suppressed. Blockade of JAK1/STAT3 signal pathway by knocking down STAT3 significantly weakened M2 polarization of RAW264.7 macrophages induced by adenosine in coculture condition and suppressed proliferation of CT26 cells, which further confirmed the effect of TAMs on tumor growth. Although our previous results couldn’t exclude the possiblity of direct impact of Atg5 and CD73 knocking down or extracellular adenosine on the proliferation of CT26 cells, this part of study indicated that factors reducing M2 polarization of macrophages indeed could inhibit the growth of CT26 cells at least partially through altering tumor immunity related to TAMs.

Different methods of fasting have been reported to be good for tumor treatment. Many studies have focused on the combination of fasting and chemotherapy or radiotherapy [10, 32–34]. D’Aronzo et al. reported that 24 h starvation could enhance gemcitabine effect in a pancreatic cancer xenograft mouse model [35]. Our study indicates that alternate day fasting is also effective in delaying colon cancer growth, providing new approach for cancer prevention and cure. It is our later work to investigate the relationship between alternate day fasting and colon cancer in human. Moreover, whether fasting is efficient for other cancer types needs further investigation.

## MATERIALS AND METHODS

### Cell culture

Mouse CT26 cell lines purchased from the TypeCulture Collection of the Chinese Academy of Sciences (Shanghai, China) and RAW 264. 7 cell lines, a kind gift of Prof Miao (Fudan University Shanghai Cancer Center, China) were both grown in RPMI-1640 medium (BI, Israel) supplemented with 2g/l glucose, penicillin/streptomycin and 10% fetal bovine serum (FBS) as normal condition. All the cells were cultured at 37°C, in a 5% CO_2_ incubator. The cells were cocultured in a transwell system at a ratio of 1: 1 to simulate the tumor microenvironment.

### *In vitro* starvation

CT26 cells and RAW264.7 cells were cocultured in RPMI-1640 with low glucose (0.5 g/l) and 1% FBS to achieve fasting condition as previously described [32]. Cells were washed twice with PBS before switching to fasting medium.

### Atg5, CD73 and STAT3 knockdown in CT26 and RAW264.7 cells

The small hairpin RNA (shRNA) targeting mouse Atg5 (shAtg5, CACCGGACTGCAGAATGACAGATTTCAAGAGAATCTGTCATTCTGCAGTCCTTTTTTG),CD73(shCD73,CACCGCAGCCTGAAGTAGATAAACTTTCAAGAGAAGTTTATCTACTTCAGGCTGCTTTTTTG),STAT3(shSTAT3,CACCGGAGCAGCATCTTCAGGATGTTTCAAGAGAACATCCTGAAGATGCTGCTCCTTTTTTG) and a scrambled shRNA used as negative control (NC, Addgene) were synthesized and cloned into the pLenti-C-Myc-DDK (Origene, China) to generate the lentirival expression vectors. For lentivirus production, 1 μg of shRNA expression plasmid and 1 μg of helper plasmids (0.4 μg pMD2G and 0.6 μg psPAX2) were transfected into 293T cells (the TypeCulture Collection of the Chinese Academy of Sciences, China) with Effectene reagent (HilyMax, Japan). The supernatants were collected 48 h after transfection and cleared through a 0.45-μm filter. CT26 cells or RAW264.7 cells were infected with viral supernatants containing 4 μg/ml polybrene (Sigma, USA) for 24 h. After the transduction, the cells were selected with puromycin (VWR, USA) for 7 days for stable shRNA expression.

### Cell proliferation assay

Cell viability was measured using cell counting kit-8 (CCK8) assay (Dojindo, Japan) according to the manufacture’s instructions. In short, CT26 was cocultured with macrophage cells as described above. Cells were grown for the indicated times. Then cells were transferred to 96-well plates at a concentration of 5×10^3^ cells/well and 10 μL of CCK8 solution was added to each well and cells were further incubated for 2 h.The optical density (OD) values were measured using a microplate reader at 450 nm.

### Mouse model and *in vivo* fasting

All animal studies and procedures were approved by the Medicine and Public Health Animal Care and Use Committee of Shanghai Medical College at Fudan University. Wild-type BALB/c mice (female, 6 weeks, 20–25 g) were purchased from Shanghai Sippr-BK laboratory animal Co. Ltd.. To establish a colon cancer bearing mouse model, 12 mice were injected subcutaneously with 100ul CT26 cells resuspended in PBS at a density of 2×10^6^ cells/mL. When tumors were palpable 5-7 days after tumor cell inoculation, the animals were divided into 2 groups. “Control” mice (N=6) with no treatment which were kept under standard conditions for the whole duration of the study; “ADF” mice (N=6) that were subjected to alternate day fasting and ad libitum diet on non-fasting days with free access to water every day. Mice were individually housed in a clean new cage to avoid cannibalism or coprophagy. Tumor sizes, body weights and general behavior were monitored every other day. Tumor sizes were measured by a caliper and tumor volume was calculated using the following equation: tumor volume (mm^3^) = (length × width × width) × π/6, expressing length and width in mm.

### Elisa

For *in vitro* experiments, the adenosine levels in supernatants of coculture system were measured with ELISA kit (Biovision, USA) according to the manufacturer’s instructions. The optical density at 450 nm each well was determined by PerkinElmermicroplate reader (Molecular Devices, USA). All experiments were performed in triplicates.

### Western blotting

Cells were harvested and lysed with lysis buffer (Beyotime, China) containing protease inhibitors (PMSF, Dingguo, China). Cellular proteins were collected after centrifugation at 10 000 × g at 4°C, and boiled for 5 min together with loading buffer. Proteins were separated by 10% SDS-PAGE, and further transferred onto nitrocellulose membranes (Bio-Rad, USA). The membranes were incubated with primary antibodies (anti-Atg5, Santa Cruz, USA; anti-LC3I/II, Santa Cruz, USA; anti-CD73, Santa Cruz, USA; anti-JAK1, Santa Cruz, USA; anti-STAT3, Santa Cruz, USA; anti-P-JAK1, Cell Signaling Technology, USA; anti-P-STAT3, Cell Signaling Technology, USA) overnight at 4°C after blocking with 5% fat-free milk in TBS-T for 1 h at room temperature. Then the membranes were incubated with a HRP-conjugated secondary antibody for 1h at room temperature followed by three-time washing with TBS-T. Bands were detected with ELL by X-ray films (MidSci, St. Louis, MO, USA) after three-time washing with TBS-T.

### qPCR

Total RNA was isolated from cells by Trizol® (Invitrogen, Carlsbad, CA). The cDNA was synthesized using iScript™ cDNA Synthesis Kit (BioRad, Hercules, CA, USA) according to the manufacturer’s instructions. Quantitative real-time PCR (qPCR) reactions were performed to detect the expression of Atg5, CD73 and STAT3 with the iTaq™ SYBR Green Supermix (Bio-Rad, Hercules, CA) and StepOnePlus™ Real-Time PCR System (Life Technologies, Carlsbad, CA, USA) according to the manufacturer’s protocol. The qPCR primers used in this study can be found in Supplementary Table S1.

### Flow cytometry

For *in vivo* experiments, tumor tissues were prepared as previously described [36–38], in brief, tumor tissues were mechanically dissociated using scalpels and after additional enzymatic dissociation with 1 mg/mL collagenases for 40 min, the fragments were filtrated through a 70 µm cell strainer. Cell suspensions were used for flow cytometry analysis. The expression of M2 macrophage biomarkers CD206 (Cell Signaling Technology, USA) and F4/80 (BD Biosciences, USA) was detected by flow cytometric analysis. For *in vitro* experiments, the expression of M2 macrophage biomarker CD206 was detected by flow cytometric analysis. In brief, cells were stained with anti-CD206 (Cell Signaling Technology, USA) for 30 min at room temperature according to the manufacturer’s instructions. Then cells were collected and analyzed. M2 polarization of macrophages was assessed by the percentage of positively stained cells. Flow cytometric data was acquired using a FACSAria™ flowcytometer (BD Biosciences, USA) and analyzed with FlowJo software version 7. 6. 1 (flowjo. com). All experiments were repeated in triplicates.

### Statistical analysis

All results were presented as mean ± SD (standard deviation). Statistical analysis was performed by t-test or one-way ANOVA with GraphPad Prism Version 5 (GraphPad Software, San Diego, CA). In all cases, difference was consideredstatistically significant at P < 0.05.

## CONCLUSIONS

**Figure 8:**
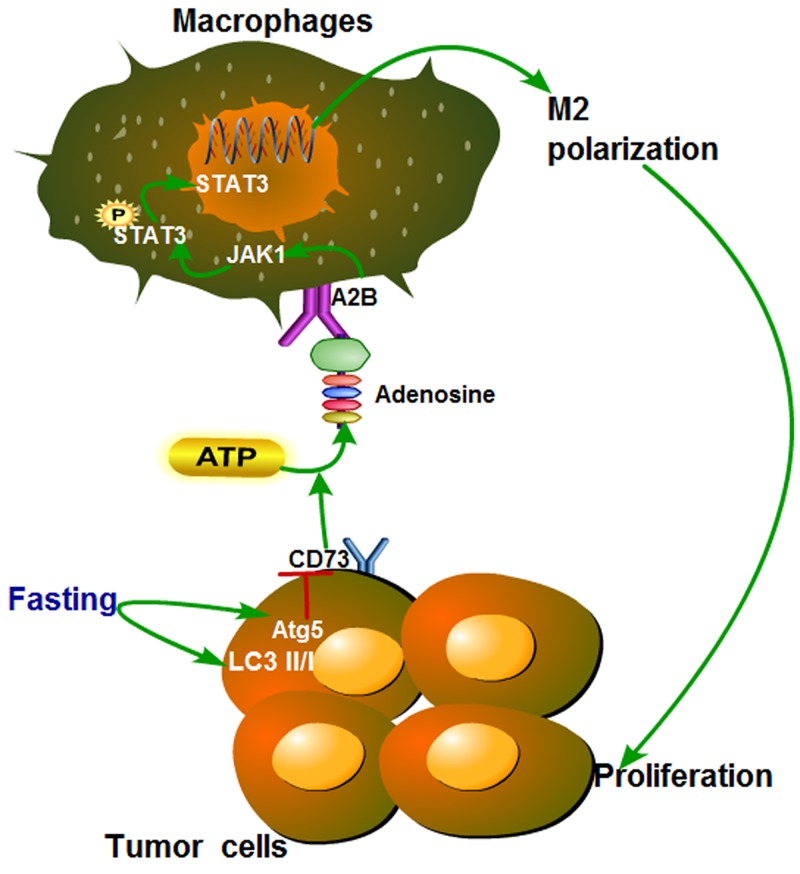
Proposed model of the effect of fasting on STAT3 pathway during polarization of tumor-associated macrophages Fasting induces expression of autophagy-related Atg5 and LC3II/I of colon cancer, then downregulates the level of adenosine by suppressing CD73, which modulates M2 polarization of TAMs through inactivating JAK1/STAT3, and eventually inhibits tumor growth.

To our knowledge, our work is the first to uncover the link between fasting and TAMs in tumor microenvironment. In summary, this study showed that fasting could induce autophagy of colon cancer cells and then downregulate the level of adenosine, which increased M2 polarization of TAMs through inactivating JAK1/STAT3, and eventually inhibitted tumor growth by promoting antitumor immunity (Figure 8) *in vitro* and *in vivo*.

## SUPPLEMENTARY MATERIALS FIGURES AND TABLE



## Abbreviations

Tregs: regulatory T cells
MDSCs: myeloid-derived suppressor cells
TAMs: Tumor-associated macrophages
JAK1: Janus Kinase 1
STAT3: Signal Transducer and Activator of Transcription 3
Atg5: Autophagy Related 5
LC3: Microtubule Associated Protein 1 Light Chain 3
HIF-1α: Hypoxia Inducible Factor 1 Alpha
MAPK: Mitogen-Activated Protein Kinase
JNK: c-Jun N-terminal kinase
PI3K: Phosphatidylinositol 3-Kinase
AKT: Protein Kinase B
