# Supplementary material for: Fasting inhibits colorectal cancer growth by reducing M2 polarization of tumor-associated macrophages
Source: Oncotarget. 2017 Aug 16;8(43):74649–60. doi: 10.18632/oncotarget.20301 (PMC5650369; doi:10.18632/oncotarget.20301)
Supplement: Supplementary file 1 [file oncotarget-08-74649-s001.pdf]

Fasting inhibits colorectal cancer growth by reducing M2 polarization of tumor-associated macrophages

SUPPLEMENTARY MATERIALS

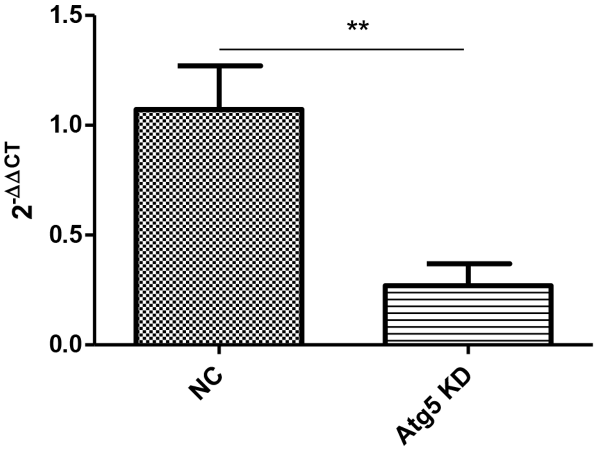

Supplementary Figure 1: CT26 cells were stably transfected with shAtg5(Atg5 KD) or shNC (NC). The Atg5 mRNA was determined by qPCR. **P<0.01.

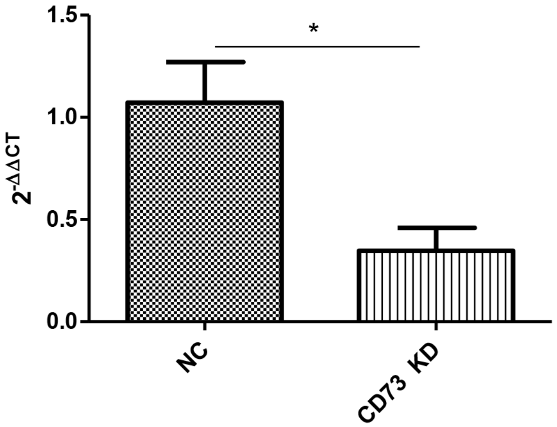

Supplementary Figure 2: CT26 cells were stably transfected with shCD73 (CD73 KD) or shNC (NC). The CD73 mRNA was determined by qPCR. *P<0.05.

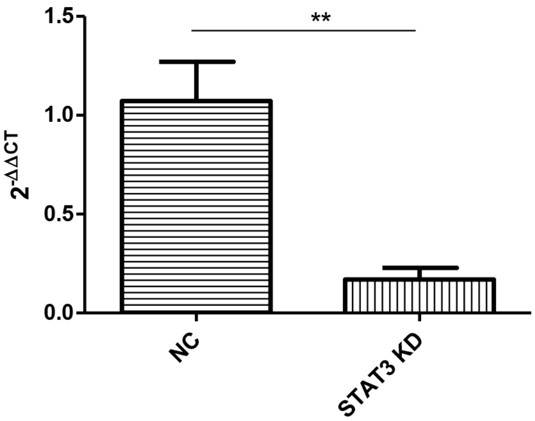

Supplementary Figure 3: RAW264.7 macrophages were stably transfected with shSTAT3 (STAT3 KD) or shNC (NC). The STAT3 mRNA was determined by qPCR. **P<0.01.

Supplementary Table 1: Primers used in this study

Gene name	Primer sequence (5′ to 3′)
mus-ATG5-F	ACCTTCTACACTGTCCATCCAA
mus-ATG5-R	GCTCCGTCGTGGTCTGATAT
mus-STAT3-F	GCGGAGAAGCATTGTGAGTGA
mus-STAT3-R	AGACGGTCCAGGCAGATGTT
mus-CD73-F	CTAGAGCAGACCAGCGATGAC
mus-CD73-R	CAGTCCTTCCACACCGTTATCA
mus-GAPDH-F	TGGTGAAGGTCGGTGTGAAC
mus-GAPDH-R	CGTGAGTGGAGTCATACTGGAA
